# Supplementary material for: A neural network model for the evolution of learning in changing environments
Source: PLoS Comput Biol. 2024 Jan 30;20(1):e1011840. doi: 10.1371/journal.pcbi.1011840 (PMC10857588; doi:10.1371/journal.pcbi.1011840)
Supplement: Attachment — Submitted filename: review report.pdf [file pcbi.1011840.s002.pdf]

Manuscript Number: PCOMPBIOL-D-23-01364

Author: Magdalena Kozielska and Franz J. Weissing

Title: A neural network model for the evolution of learning in changing environments

Exploring the evolutionary process of learning based on neural networks has been drawing great attention since long. In this work, the authors introduced one intriguing factor, that is, environment changing, into the baseline model of a biology-inspired learning mechanism. They showed the evolutionary outcomes both with and without fixed learning periods. Furthermore, they also investigated the impact of environment quality distribution and lifespan parameters upon the evolutionary dynamics of the learning process. Overall, I think this topic is interesting and important, and the proposed modeling framework is, as far as I know, original. The results within the main text and supplementary information are pretty comprehensive. However, I have some concerns and suggestions on the current manuscript that should be addressed first.

1. With due respect, I suggest the authors partially rewrite the abstract in order to highlight their main results. From my perspective, the current abstract does not provide a clear and straight-forward statement of the findings. I also found it difficult to dig out the corresponding gap and significance of the work from this abstract. Reducing the number of brackets by taking out or deleting the contents and adding 1-2 sentences that briefly introduce the background of this field and their motivations at the beginning should do the trick.

2. In the first paragraph of introduction, the authors should involve relative literatures to support the background discussion, i.e., 'Learning has been studied extensively in different fields, like psychology, ethology, neurobiology, and more recently artificial intelligence.' Also, there should be more references involved in the next sentence, i.e., 'A number of theoretical studies have been conducted in order to understand the evolution of learning but many questions still remind unanswered.'

3. This model contains quite a few parameters and statistical magnitudes. In order to make the results more readable, I suggest the authors list out the notations with their explanations in the Method part.

4. What is the effect of learning rate? Also, another concern over the usage of notations: The authors represented learning rate with 'L' in equation 2, while this notation only appeared once in the text below. This may cause an inconvenience situation to understand the model and the results.

5. Since the generation of neural networks is random, where the initial weights and

biases are drawn from the uniform distribution, would 20 replicates for each set of parameters be enough to reflect the whole picture? I notice that in some panels (situations of f=0.01 in Fig 8 and 9, for instance), the distribution patterns of the spots may not be fully revealed. Increasing the number of replicates for specific cases can probably be helpful.

6. Fig 3 and Fig 7 should be replaced with a clearer version.

7. Results and tendencies in the results of Fig 5 (Figure 5) is not as clear as the text expressed, since in some cases the innate prediction profile also performed quite well. Can the authors provide a quantified results comparison?

8. I believe the sentence 'Interestingly, when…' on line 485 is describing the results from Fig 9, while it is located in a paragraph starting with 'Fig 10'. I suggest the authors reorganize the paragraphs discussing the results referring Fig 9 and 10 (from line 474 to 488) for a more logical narrative.

Some minor comments:

1. In line with other captions, captions of Figure 5 and Figure 9 should be Fig 5 and Fig 9.

2. Line 247, please use a notation for the parameter lifespan, instead of writing mathematical expressions like '[0, lifespan]'.

3. Line 252, please use fifty thousand or 50,000 instead of 50 thousand.
