# Supplementary material for: A neural network model for the evolution of learning in changing environments
Source: PLoS Comput Biol. 2024 Jan 30;20(1):e1011840. doi: 10.1371/journal.pcbi.1011840 (PMC10857588; doi:10.1371/journal.pcbi.1011840)
Supplement: Attachment — Submitted filename: 2023-12-18 R2R letter.docx [file pcbi.1011840.s003.docx]

Response to the reviewers

"A neural network model for the evolution of learning in changing environments"

Manuscript Number: PCOMPBIOL-D-23-01364

We thank the reviewers for their constructive comments. Before addressing these comments in detail, we briefly summarise the main changes made to the manuscript.

- In response to Reviewer 1, we added a justification of why we considered only between-generation environmental change to the Methods section (lines 217-222 of the clean version of the revised manuscript) and we discuss some implications of this assumption in the Discussion section (lines 640-652).
- In response to Reviewer 2, we edited the abstract, included more references to the literature, added a table with the main model parameters, their interpretation, and the range of values studied, doubled the number of replicates per parameter combination, and updated all figures.
- To improve the readability of the manuscript, we improved the order of presentation and/or the phrasing in various locations in the manuscript.

Reviewer 3 correctly summarised our work but did not express any concerns. Therefore, we do not consider it necessary to address the reviewer’s report.

Reviewer 1:

Generally speaking, I am really enthusiastic about the work presented in this MS. As the authors point out there are relatively few formal models of the evolution of learning and none that explicitly consider how network-based learning is likely to evolve. In addition, the analyses are well executed and clearly discussed, and so the work will almost certainly have an impact on the field. I have only one main issue I feel needs to be explicitly considered in the MS to maximise its potential.

Most of the functional ("behavioural gambit") logic underpinning existing understanding about the selective advantages of learning emphasise the key role of within generation (within life time) patterns of variation with the received wisdom being that learning is most strongly selected for when within-generation variation is substantial, varies between generations and potentially trackable. However, the authors only consider ecological change happening between generations here. That learning is still favoured at all seems to me to be because there is a noisy cue that can be at least partially resolved via repeated sampling. Now none of this renders the analysis uninteresting - the selective value of being able to resolve such cue error via learning is not something that has been widely considered in the evolutionary literature to my knowledge. Nevertheless, i do feel that the authors need to acknowledge this issue and discuss how their formulation relates to such "classic" ideas about how ecological change affects the evolutionary value of learning. This received wisdom is derived from Dave Stephens' early discussions and analyses culminating in his seminal model in Stephens (1991), which I note the authors do not cite.

Thanks for pointing this out! We agree that we did not address the time structure of environmental change (and the implications of the chosen structure) in sufficient depth and detail. In the revised version, we now state clearly that within-generation environmental change will typically lead to “life-long learning”, while our model assumes that learning takes place at the start of an individual’s life. We also indicate that in the context of within-generation change one should allow for the evolution of more sophisticated learning strategies (“learning schedules”), which we postpone to a future attempt.

However, we do not agree with the reviewer's interpretation of Stephens’ 1991 article. Stephens did pioneering work regarding the adaptive value of learning, and his 1991 article is an important contribution. But in our view, this article does not support the conclusion that within-generational environmental change is crucial for learning to evolve. By means of a simple model, Stephens showed that, contrary to verbal arguments, “learning *can* evolve when there is no within-generation persistence” (Stephens 1991, p. 84, our emphasis). However, he also concluded that “There are no conditions in which learning is more likely to evolve than when between-generation persistence is low and within-generation persistence is high” (p. 84) – the case studied in our model. In the Abstract, Stephens also states “(1) The change required to promote the evolution of learning can occur either within or between generations even though (2) the regularity required to promote the evolution of learning must come within an animal’s lifetime.” Our model considers the case with high regularity/ persistence within generations and different degrees of change between generations. One could therefore conclude that our model represents the best-case scenario for the evolution of learning considered by Stephens. However, one should not over-interpret Stephens’ conclusions. With the methods available in 1991, Stephens had to consider an extremely simple model with only two choices (one with fixed and one with variable outcomes) and a simple “learning rule”, which represents a fixed decision strategy. Our model considers a more complex environment (with a broad spectrum of resources) and a more challenging learning task (predicting the current environmental profile). It is therefore not self-evident that Stephens’ conclusions apply to a model like ours.

We agree with Reviewer 1 that most behavioural ecology models on the evolution of learning tend to focus on (temporal or spatial) within-generation variation in environmental conditions. Presumably, this focus is related to the desire to test the model predictions in an experimental setting. Interestingly, other branches of evolutionary biology focus on between-generation change when studying the evolution of learning (e.g. Aoki et al. 2025, Aoki and Feldman 2014 and references therein). Obviously, both types of models are well-established in the literature.

It would have been interesting to discuss all this in our manuscript, but we have the impression that this would lead us astray from our main goal: to present a more mechanistic model for the evolution of learning when there is a trade-off between exploration (learning) and exploitation (foraging). In this context, it is convenient to separate the exploration and the exploitation phase in time by assuming that individual life consists of a learning phase early in life followed by a foraging phase later in life. Such an assumption makes perfect sense if the environmental characteristics remain stable throughout the lifetime of an individual: in such a case, it is optimal to learn these characteristics as early as possible and to profit from the learned insights when foraging. We now justify our modelling choice in the Methods section (217-222 of the clear version of the revised manuscript) and come back to this in the Discussion (lines 640-652). There, we indicate that (1) our model can readily be applied to contexts involving within-generation environmental change, but that (2) this would mainly make sense if more complex learning schedules are considered.

Reviewer 2:

Exploring the evolutionary process of learning based on neural networks has been drawing great attention since long. In this work, the authors introduced one intriguing factor, that is, environment changing, into the baseline model of a biology-inspired learning mechanism. They showed the evolutionary outcomes both with and without fixed learning periods. Furthermore, they also investigated the impact of environment quality distribution and lifespan parameters upon the evolutionary dynamics of the learning process. Overall, I think this topic is interesting and important, and the proposed modeling framework is, as far as I know, original. The results within the main text and supplementary information are pretty comprehensive. However, I have some concerns and suggestions on the current manuscript that should be addressed first.

1. With due respect, I suggest the authors partially rewrite the abstract in order to highlight their main results. From my perspective, the current abstract does not provide a clear and straight-forward statement of the findings. I also found it difficult to dig out the corresponding gap and significance of the work from this abstract. Reducing the number of brackets by taking out or deleting the contents and adding 1-2 sentences that briefly introduce the background of this field and their motivations at the beginning should do the trick.

Thanks for pointing this out. In view of the length limitations, we earlier removed a sentence that placed our work in the context of the current literature. We hope that the new version of the Abstract clarifies matters.

2. In the first paragraph of introduction, the authors should involve relative literatures to support the background discussion, i.e., ‘Learning has been studied extensively in different fields, like psychology, ethology, neurobiology, and more recently artificial intelligence.’ Also, there should be more references involved in the next sentence, i.e., ‘A number of theoretical studies have been conducted in order to understand the evolution of learning but many questions still remain unanswered.’

Thanks again, we fully agree. We have now added the requested references.

3. This model contains quite a few parameters and statistical magnitudes. In order to make the results more readable, I suggest the authors list out the notations with their explanations in the Method part.

We added a new table (Table 1) that lists the key parameters of the model, briefly explains their meaning, and indicates the parameter range investigated. When doing this, we realised that we introduced some symbols that are used only once (in the Methods section). Instead of adding all these symbols to the table, we removed them from the text, which we believe makes the Methods easier to read. Symbols referring to the functioning of the neural network (*w*, *b*, *x*, *y*) are retained in the text as they are necessary to order to understand the equations. However, they are not added to the table as they are not needed to understand the simulations and the results. Including these symbols would, in our opinion, make Table 1 unnecessarily large and more difficult to read.

4. What is the effect of learning rate? Also, another concern over the usage of notations: The authors represented learning rate with ‘*L*’ in equation 2, while this notation only appeared once in the text below. This may cause an inconvenience situation to understand the model and the results.

We added additional explanation of the learning rate in lines 177-179 of the clear version of the revised manuscript: “*L* is the learning rate, a heritable factor that determines how strongly the network weights are modified during each learning event. For a given prediction error, a higher learning rate leads to larger change in the four network weights affected by learning.”

We wanted to show the mathematical equation for the Delta rule as it is an important ingredient of our model. Therefore, we introduced a symbolic notation to represent the learning rate in the equation (*L*). It is correct that we used this notation only once more in the text. We believe that adding it in more places would make the text more difficult to read while not adding to the understanding. For consistency, we no longer mention the symbol *L* in line 245.

5. Since the generation of neural networks is random, where the initial weights and biases are drawn from the uniform distribution, would 20 replicates for each set of parameters be enough to reflect the whole picture? I notice that in some panels (situations of f=0.01 in Fig 8 and 9, for instance), the distribution patterns of the spots may not be fully revealed. Increasing the number of replicates for specific cases can probably be helpful.

As suggested by the Reviewer, we run more simulations for the scenarios where the number of learning episodes evolves. New figures with 40 replicates (instead of the original 20) for each environmental condition are now placed in the revised manuscript and the supplementary material. Doubling the number of replicates confirmed our earlier conclusions but was indeed helpful to fully reveal the distribution of simulation outcomes. A few small remarks were made to the text (lines 446-447 and 462 of the clean version of the revised manuscript).

6. Fig 3 and Fig 7 should be replaced with a clearer version.

Higher-quality versions of all figures were made for the revised manuscript.

7. Results and tendencies in the results of Fig 5 (Figure 5) is not as clear as the text expressed, since in some cases the innate prediction profile also performed quite well. Can the authors provide a quantified results comparison?

The difference between the environmental profile and the prediction profile can indeed be quantified by calculating the integral of the absolute differences between the functions to be compared. We did this for all examples illustrated in Fig 5. With one exception (the fourth example for the case of 5 learning episodes), the difference between the prediction profile and the environmental profile was larger before learning than after learning. In the exceptional case, the calculation confirms the visual impression that the prediction profiles before and after learning are almost identical.

As Fig 5 mainly serves an illustrative purpose, we decided not the add these quantitative results to the manuscript. That learning leads to a better “prediction” (or “understanding”) of the environment can also be concluded from the fact that learning (if it evolves) leads to a higher performance than the innate prediction profile (as shown in Fig 3).

8. I believe the sentence ‘Interestingly, when…’ on line 485 is describing the results from Fig 9, while it is located in a paragraph starting with ‘Fig 10’. I suggest the authors reorganize the paragraphs discussing the results referring Fig 9 and 10 (from line 474 to 488) for a more logical narrative.

Good point. We moved the section starting with “Interestingly, when…” to the previous paragraph (lines 504-508) where it indeed fits better.

Some minor comments:

1. In line with other captions, captions of Figure 5 and Figure 9 should be Fig 5 and Fig 9.

This has been corrected.

2. Line 247, please use a notation for the parameter lifespan, instead of writing mathematical expressions like ‘[0, lifespan]’.

To avoid adding an extra symbol for a lifespan that is not used anywhere else we decided to rephrase this part of the sentence to “the length of the learning period is an integer ranging from zero to the lifespan of the organism.” (lines 246-247)

3. Line 252, please use fifty thousand or 50,000 instead of 50 thousand.

Corrected.
